# GPCRs Are Optimal Regulators of Complex Biological Systems and Orchestrate the Interface between Health and Disease

**DOI:** 10.3390/ijms222413387

**Published:** 2021-12-13

**Authors:** Hanne Leysen, Deborah Walter, Bregje Christiaenssen, Romi Vandoren, İrem Harputluoğlu, Nore Van Loon, Stuart Maudsley

**Affiliations:** 1 Receptor Biology Lab, University of Antwerp, 2610 Wilrijk, Belgium; hanne.leysen@uantwerpen.be (H.L.); deborah.walter@student.uantwerpen.be (D.W.); bregj.christiaenssen@student.uantwerpen.be (B.C.); romi.vandoren@student.uantwerpen.be (R.V.); irem.harputluoglu@metu.edu.tr (İ.H.); nore.vanloon@student.uantwerpen.be (N.V.L.); 2 Department of Chemistry, Middle East Technical University, Çankaya, Ankara 06800, Turkey

**Keywords:** dimensionality, G protein-coupled receptor, network, pharmacology, precision, quantitative, therapeutic, DNA damage, allostasis, systems biology

## Abstract

GPCRs arguably represent the most effective current therapeutic targets for a plethora of diseases. GPCRs also possess a pivotal role in the regulation of the physiological balance between healthy and pathological conditions; thus, their importance in systems biology cannot be underestimated. The molecular diversity of GPCR signaling systems is likely to be closely associated with disease-associated changes in organismal tissue complexity and compartmentalization, thus enabling a nuanced GPCR-based capacity to interdict multiple disease pathomechanisms at a systemic level. GPCRs have been long considered as controllers of communication between tissues and cells. This communication involves the ligand-mediated control of cell surface receptors that then direct their stimuli to impact cell physiology. Given the tremendous success of GPCRs as therapeutic targets, considerable focus has been placed on the ability of these therapeutics to modulate diseases by acting at cell surface receptors. In the past decade, however, attention has focused upon how stable multiprotein GPCR superstructures, termed receptorsomes, both at the cell surface membrane and in the intracellular domain dictate and condition long-term GPCR activities associated with the regulation of protein expression patterns, cellular stress responses and DNA integrity management. The ability of these receptorsomes (often in the absence of typical cell surface ligands) to control complex cellular activities implicates them as key controllers of the functional balance between health and disease. A greater understanding of this function of GPCRs is likely to significantly augment our ability to further employ these proteins in a multitude of diseases.

## 1. Introduction

### 1.1. G Protein-Coupled Receptors

It is reasonable to contend that the most therapeutically important molecular targets at the present time are the transmembrane heptahelical G protein-coupled receptors (GPCR). GPCRs are the largest family of transmembrane receptors in humans and many other species and represent the most diverse family of targets for current therapeutics [1,2,3,4]. GPCRs facilitate communication between cells in tissues across long distances in the body, thereby enabling the capacity for system-level therapy [5,6,7,8]. Therapeutics effectively exploited GPCR systems many years even before the discovery of GPCRs themselves [9,10]. Controllers of these receptors were originally designed to exert either a simple positive effect (increasing the activity of downstream signaling systems, e.g., adenylate cyclase) or by inhibiting this activity by occupying the receptor and antagonizing the positive actions of stimulatory ligands. Therapeutic agents were classified as simple agonists or antagonists based on the concept that receptors could exist predominantly in two distinct states, i.e., inactive and active.

Work from multiple talented laboratories nearly three decades later largely confirmed this two-state model for GPCRs [11,12,13,14,15,16,17]. With the introduction of molecular alterations to GPCRs [18], it was demonstrated that GPCRs indeed exist in a spontaneous equilibrium between two conformations, i.e., active (R*) and inactive (R). The active conformation is stabilized by agonist binding or by mutagenesis that can relieve intramolecular constraints [18,19,20,21,22,23]. In this model, GPCRs transmit signals through their capacity to act as guanine nucleotide exchange factors for the heterotrimeric guanine nucleotide-binding proteins (G proteins) in response to stimulatory ligand binding. G protein activation is initiated through conformational rearrangement of the GPCR transmembrane core and juxtamembrane loop regions, eventually catalyzing the exchange of GDP for GTP on the receptor-associated Gα subunit [24,25,26,27,28]. A guanine nucleotide exchange (GDP for GTP) then initiates the dissociation of the heterotrimeric G protein from the GPCR, followed by the break-up of the G protein heterotrimer releasing free GTP-bound α and βγ subcomplexes. These two signaling components can stimulate, inhibit or physically recruit multiple downstream signal transduction effectors, e.g., adenylyl cyclase (AC), phospholipase C (PLC), GPCR kinases (GRKs) or GRK-interacting proteins [29]. In this manner, the heterotrimeric G protein can transmit information to the intracellular milieu about the qualitative and quantitative nature of a specific extracellular stimuli [30,31].

### 1.2. Signaling Diversity in GPCRs 

#### 1.2.1. G Protein and Non-G Protein Signaling 

Since their discovery, GPCRs have been considered to be primarily G protein-signaling entities. This knowledge has been demonstrated to be exceptionally successful in allowing the creation of a huge variety of effective pharmacotherapeutics. Hauser et al. evaluated in 2017 that 475 FDA-approved drugs target GPCRs, which is 34% of all FDA-approved drugs [32]. Even with this specific G protein focus, agents have been generated that can control the bias amongst diverse forms of G protein-signaling output [23,33]. In the last decade, our appreciation of GPCR-signaling complexity has been enhanced by the demonstration of simultaneous signaling activities emanating from GPCRs that are either G protein-based or controlled by non-G protein-signaling adaptors. Even with just the primary consideration of G protein activation, it is evident that the receptor conformations for G protein activation are different between specific G protein pools and that synthetic and naturally occurring ligands can selectively facilitate the formation of different receptor coupling conformations [23,34]. Multiple distinct forms of agonist ligands for a single GPCR type have now been discovered to only activate a subset of G proteins or a subset of downstream signaling effectors or induce G protein coupling without initiating internalization and desensitization [23,35,36]. Given the successful exploitation of the therapeutic intervention of GPCR-based G protein signaling, it is likely that it will be possible to improve this index even further by exploiting the true complexity and diversity of GPCR signaling [32,37]. Apart from the classical G protein signaling, multiple research lines have pointed towards the presence of non-G protein-based signaling, mainly through β-arrestins [38,39,40]. One of the first studied examples of this novel signaling activity was the β-arrestin-dependent activation of extracellular signal-regulated kinases 1/2 (ERK 1/2) [29,41]. Compared to the rapid and transient manner of G protein signaling [39], β-arrestin-linked pathway activation has a later onset but is sustained over a long period and entrains long-term l cellular transcriptional and proteomic effects [42,43,44,45]. β-arrestin also serves as a negative regulatory protein for signaling through G proteins and is responsible for GPCR internalization [45,46,47]. However, there is still some dispute in the field with respect to the interdependence of G protein and β-arrestin signaling; recently, complete G protein independence could not be proven in a serum-starved in vitro G protein knockout model [41]. Evidence has also been revealed recently that suggests that certain specific forms of orphan receptors (D6R and C5aR2) appear to be able to functionally interact with β-arrestins but not with G proteins [48]. This data reinforces the posit that there are likely a diverse range of receptorsome entities that are prewired to specific independent downstream signaling pathways.

#### 1.2.2. G Protein-Coupled Receptor Complexes

Since 1999, it has become clear that GPCR signaling is more complex, specific and diverse than initially considered in the two-state model [28,49]. One of the factors accounting for this complexity is the ability of receptors to form multistate-signaling complexes or so-called receptorsome structures. These receptorsome structures comprise the receptor itself combined with multiple interacting proteins. These preassembled receptorsomes demonstrate unique pharmacology, signaling, trafficking, desensitization and internalization features [50,51,52,53]. This conditioning of GPCR activity is also influenced by the relative variations of these adaptor proteins in distinct tissues, suggesting the presence of tissue-specific GPCR activity [23,37,54]. Given this growth in GPCR-signaling complexity, it is vital to recognize the unique properties of endogenous or cognate ligands for GPCRs. The proposed cognate ligands of GPCRs attempt to impact every consequence of receptor activation in the same manner, whether desensitization, internalization, trafficking or G protein coupling [43,55]. Hence, these ligands strive to engender an omnipotent efficacy. The ability of a ligand to achieve this at a systemic multi-tissue level is, however, highly unlikely due to tissue-to-tissue variations in the receptor and signaling adaptor expression under the influence of diverse cellular conditions [23,56,57,58,59]. Given the ability for multiple ligands to stimulate the same GPCR, it may be prudent to redefine our conceptualization of endogenous ligand cognation. Indeed, the most accurate definition of a cognate ligand for a specific GPCR could be codified by its ability to most equally regulate the full GPCR-signaling spectrum across a diverse series of tissue/cell settings. Hence, it is likely that receptors and their cognate ligands coevolved to elicit the most physiologically adaptive and effective responses in target cells. With respect to the concept of functional relationships between cognate ligands and their preferred receptors, the aging/stress response paradigm presents an important pathophysiological process that represents perhaps the greatest systemic and coordinated perturbation of cellular signaling in human physiology. 

This complexity and diversity of GPCR signaling also reveals the necessity to change our definition of agonists and antagonists originally based on the two-state model [18,19,20,21,22]. Ligands, to varying degrees, likely stabilize these different active conformational states and thereby initiate diverse signaling pathways. This concept is known as biased agonism. Verifying and understanding this mechanism will likely help to develop functionally selective drugs, which activate beneficial downstream pathways and suppress adverse side effects [23,24]. 

It is evident that an in-depth understanding of the effective G protein-coupling capacity of GPCRs has been effective for the development of GPCR-based therapeutics [60]. The G protein-centric focus of GPCR signaling was expanded by the discovery that β-arrestins—originally thought of just as terminators of G protein signaling [61,62]—can also act as productive signaling effectors [49]. Further research has demonstrated that the realm of GPCR signaling is far more complex and diverse than initially imagined [2,37,43]. This signaling diversity arises from several factors associated with subcellular localization, specific post-translational modification states of the receptor and, perhaps most importantly, the ability of the receptor to exist in multiple receptorsome-signaling states. In multistate signaling GPCR models, specific agonists likely possess the ability to activate distinct active receptorsomes by exposing different intracellular regions involved in coupling separate G protein pools, initially demonstrated for the β2-adrenergic receptor antagonist ICI-118-551 [63,64], and β-arrestin signaling [45,49]. It is becoming more evident each year that agonist-selective receptor signaling, targeting a subset of the possible response profiles, may represent an opportunity to develop drugs that are more precise and could also have an increased efficacy. 

To assist the capacity to investigate these GPCR receptorsomes, there have been considerable advances in the accuracy and selectivity of proteomics-focused mass spectrometers that can investigate the specific protein stoichiometries in these complexes. This increase of protein detection sensitivity has enabled the experimental transition from whole-tissue/cell investigation to allow an analysis of protein–protein interactions at a subcellular level, i.e., interactomics [65]. The dynamic investigation of how multiprotein complexes alter in quantity and quality in response to drug exposure has enabled the examination of the subcellular network functionality of therapeutic agents [66,67,68,69]. The importance of GPCR interactomics lies in the posit that the local context of protein associations/interactions is often more strongly linked to the biological activity of a certain form of signaling pathway or disease process, as opposed to simple global cellular or tissue protein expression levels [68,70]. 

#### 1.2.3. G Protein Signaling, Endocytosis and Cellular Location

The canonical model of GPCR signaling, either through G proteins or non-G protein adaptors, has been largely considered to emanate from the plasma membrane localized receptors. One potential mechanism contributing to the diversity and specificity of GPCR signaling is through membrane trafficking and alternative residential and signaling sites of the receptor [71,72,73]. Until recently, the cell surface plasma membrane trafficking of GPCRs was considered as a mechanism to control the sensitivity to an extracellular stimulus by changing the receptor level through ligand-mediated endocytosis or reduced trafficking to the plasma membrane of newly synthesized receptors [74]. It has recently been demonstrated that GPCRs can signal from intracellular membranes such as endosomes, mitochondria, the endoplasmic reticulum, the Golgi apparatus and the nucleus [75,76]. This shift in signaling location was first identified on Gαs-coupled receptors such as the parathyroid receptor, thyroid-stimulating hormone receptor and the β2 adrenergic receptor, where cyclic adenosine monophosphate production was still evident after endocytosis [77]. Given the subcellular variations of GPCR adaptor protein expression, it is highly likely that the subcellular location of GPCR receptorsomes can assist in defining specific ensembles of adaptor-directed specific signaling activity [43].

## 2. Complex Biological Systems

Complex systems, consisting of hundreds to millions of distinct entities, typically possess underlying deterministic properties that can lead to unpredictable patterns [78]. At a rudimentary level, complex systems can be conceived as a network of multiple units that interact with each other in a nonlinear manner [79]. The relationship(s) between the different nodes can be modeled in the most basic manner by a graph. Graph theory is used to describe mathematical structures in which vertices (i.e., nodes) are connected by edges (i.e., lines) [80]. For many years, biological systems have been represented as complex sets of binary interactions between biological entities [81]. In nonlinear systems, the dynamic behavior can change dramatically when a certain parameter crosses the critical point at so-called bifurcation points [82]. In a biological context, this could be related to specific stress parameters or persistent alterations in signaling molecules. The behavior of the network indicates an underlying order (with the goal of maintaining homeostatic health), but it is difficult to predict the global behavior by knowing the input and the network components. Having to contend with coordinated and coincidental interactions between multiple nodes in a network is clearly problematical for standard research approaches, hence the recent trend for the introduction of machine-assisted inference in biomedical science [83,84]. In the field of machine learning (ML), complex systems can be compared to the concept of a neuronal network, which is a so-called black box model. It may be able to provide predictions of activity, but how the model comes to the results is often not fully comprehensible. To contend with this deficiency, a recent development for the ML-based interpretation of complex network data lies within the realm of artificial neural network–based deep learning (DL) [85]. DL allows multilayered processing models to learn representations of data with multiple levels of abstraction. DL can uncover intricate structures within large datasets by using a backpropagation algorithm [86]. Such algorithms indicate how a machine should change its internal parameters, which are used to compute the representation in each layer, from the representation in the previous layer to uncover the subtle structural properties of the data. DL-based algorithms have demonstrated superiority over most other techniques in diverse biomedical fields, such as predicting the effects of mutations in noncoding DNA on gene expression and disease [87], image recognition [88], reconstructing brain circuits [89], mass spectrometry-based proteomics [90] and, most pertinently, drug molecule activity prediction [91].

The theory of complex systems has been applied effectively to the interpretation of a wide variety of fields, such as economics, meteorology and the domain of systems biology [92,93]. It has been considered that biological systems are among the most complex to grasp and predict [94]. The equilibrium of a species within an ecosystem, the interaction of neurons and crowd behavior have all been modeled as complex systems [78,95,96]. In pharmacology, therapeutic targets can range from single atomic structures, such as metal ions controlling ion channels, to macromolecules such as proteins, whole metabolic pathways, cells and organ systems that coordinate whole organisms from a systemic signaling standpoint. Human physiology is a tightly regulated system that maintains equilibrium throughout the individual’s lifetime. Multiple factors influence the daily maintenance of the biological system and cooperate to achieve a state of equilibrium despite the randomized presence of stressful factors that disrupt the system and can lead to disease signature generation [67,83,84]. However, from a human physiological standpoint, there is not only one state of equilibrium. Every physiological disruption/perturbation can lead to a further shift towards a new equilibrium state, and eventually, the equilibrium is shifted to the point where it allows the presentation and homeostatic preservation of disease symptoms. To maintain system homeostasis, feedback loops are necessary to actively maintain a systemic balance and enable a complex cellular life. For example, the human organism possesses a precise regulation of ATP balance with the glucose-dependent regulation of insulin secretion from beta cells [97,98,99]. For highly regulated and physiologically critical systems, such as the insulinotropic system that manages the balance between glucose and insulin levels, the organism has excellent tools in the form of receptors to sense slight perturbations and to react to the changes immediately. A long-term homeostatic balance sustains the lifespan control and is underpinned by dynamic microscale feedback loops controlled by scale-adapted allostasis [100,101,102,103]. Here, we contend that disease-free aging is strongly associated with the capacity of cells to most efficiently sense random stressful perturbations and then ameliorate those effects to revert the system back to a healthy homeostasis. To support this mechanism, we propose that a coherent and dedicated network of intracellular GPCRs underpins a single-cell intrinsic stress response network that, in turn, protects against age-related disease generation.

### 2.1. Functional Properties of Complex Systems

Most complex systems of various types share some common fundamental properties of a network communication. Features such as randomness and order exert potent effects upon the behavior of local small circuits within the overall network, as well as the whole system itself [82]. In large networks, the connectivity of the nodes follows a scale-free power–law distribution that arises from the fact that new nodes preferentially connect to already well-connected locations [104]. Other properties of dynamic complex systems include the small world property, which describes the phenomenon that networks are highly clustered but, at the same time, have small characteristic path lengths, like random graphs. This organizational system increases the signal-propagation speed, computational power and synchronization [105]. Complex systems are often described as being on the edge of chaos, a transition point between order and randomness [106]. Thus, complex systems can exhibit predictable behaviors for a certain period and suddenly undergo major changes after only minor perturbations to the system. This scenario, in fact, demonstrates a potentially important property needed for a cellular stress responsive system that can rapidly readjust to the very first signs of cell stress to reduce the spread and functional impact of deleterious perturbations.

In addition to these archetypical features, it has been demonstrated over recent decades that, even though based on small and predictable events at the microscale, complex systems often demonstrate the generation of emergent properties that initially appear unrelated to the nature of the base entities forming the system. The emergent properties of a system are most apparent when the interaction between the comprising units is observed in the larger whole (e.g., at the systemic homeostatic level) and not just in their individual parts (e.g., binary protein–protein interactions). Understanding the dynamic nature of a complex network system is key to describing the physiological systems it monitors, regulates and controls. Current technologies and methods in life science that can create high-dimensionality datasets, e.g., in genomics, proteomics and transcriptomics [83,107,108], make an effective appreciation of highly complex biological networks feasible for many laboratories. These technologies unlock significant opportunities to appreciate a high percentage of all the individual constituents of complex systems simultaneously [93]. Such an in-depth understanding of systemwide alterations will probably lead to an augmented view of biological complexity that will help reveal important new functional associations. Approaching biology and disease pathology in the context of GPCR sensory systems, with the concept of a network governed by universal laws, will likely augment our understanding of these systems and help generate novel therapeutic intervention strategies [109]. Disease susceptibility (e.g., Alzheimer’s disease) is often not just the result of a single gene mutation but rather a disruption in the network context of a gene and is often more related to a subnetwork of factors linked to processes (e.g., metabolic disruption leading to Alzheimer’s disease [110]) that may be perturbed in a persistent manner. To fully appreciate the true nature of physiological functions, it has been suggested that network science should become embraced earnestly as one of the most effective pillars of molecular biological inference [111]. The network concept can be used to create a theoretical graphical model of the structure and flow of functional information in biological systems [112]. Recently, computational approaches have been applied to analyze systemwide signaling pathways after a SARS-CoV-2 infection to determine the virus-host interactions and understand the systemwide response following infection [113,114]. 

Human physiology is likely coordinated through a tightly regulated combination of multiple emergent systems. The considerable average human life expectancy of about 80 years in industrialized countries is a definitive sign of this well-functioning systemic homeostasis. This organismal stability has evolved over billions of years through nuanced stress sensory mechanisms, compensatory feedback loops and potentially perturbagen prediction systems based on GPCR-associated damage management networks [115]. Nevertheless, the random and rapid nature of stressful cellular perturbagens can often overwhelm the stress responsive network, as the protein interactome-based sensory network may only be able to respond at a slower rate due to the need to either generate ne novo proteins or reassemble intricate protein complexes. If the stress responsive network cannot fully prevent/repair the damage caused by a perturbation event, then the damage may be compounded when successive damaging perturbations are experienced by the cell [76,116,117,118,119]. Hence, in a similar manner to the process of repetitive muscular calcium loading inducing a tetanic contraction, unrepaired damage will accumulate and drive the feedforward aging process. The specific initiation point of multiple diseases may therefore indeed be created by a generic start point, i.e., the repetitive damage tetanic process, but then diversify with time to create differential end-stage disease states and symptoms [83,120,121,122].

Allostatic adjustments, which are made to maintain global homeostasis after small disruptions, are associated with a branching chain reaction or, more generally, a positive feedback loop. Systems with positive feedback loops are known to initially amplify small disturbances gradually to the point of instability and destruction of the system in the absence of negative feedback loops. An important example of such an amplification of initial small disturbances through positive feedback loops (vicious cycles) leading to the destruction of physiological systems is the pathological aging process [120]. Aging is likely orchestrated by feedback loops and the counter cycling that repeatedly repairs damage. The maintenance of homeostasis in this complex network has been demonstrated to be an indicator of healthy aging [123,124,125,126]. Increasing attention has been paid to the disease progression of patients with more than one disease to investigate the network dynamics on a system biology level. Aging-associated comorbidities can reveal additional interactions between molecular levels and external factors such as lifestyle and diet [127,128,129]. The goal of understanding the complex networks that control physiological-to-pathological trajectories is to steer them in a nonpathological direction. Structural controllability based on graph theory makes it possible to identify a minimal number of key factors for the optimal control of complex networks [130]. For effective therapeutic interventions that can restore genomic, proteomic and endocrine homeostasis, a nuanced system pharmacology approach will be needed that identifies both rational polypharmacological agents as well as combination therapies [131]. In these scenarios, GPCRs are optimal targets due to their ability to control diverse physiological functions and their ability to create stress-sensitive signaling systems and complexes [132]. Understanding how to regulate, using a multidimensional GPCR-based approach, and reverse the pathological loss of systemic homeostasis across the lifespan is vital to the future design pipeline of therapeutics that can act to suppress the generation of fully matured disease states [133].

#### 2.1.1. Networks in Pharmacological Systems

From a traditional pharmacological standpoint, the therapeutic actions of drug activity are believed to be a direct response due to the stimulation of a single index (e.g., cAMP accumulation) or the activation of signal transduction pathways. Although these views are quite limited, a single index analysis and basic enzyme pathway analysis facilitated the creation of many currently used agents that are effective in controlling disease symptoms. However, disease progression is likely a combination of complex multidimensional processes, and the therapeutic effects of certain drugs might, in fact, be collateral to the observed changes in the symptoms [83,134]. Both drug responses and different disease states can be multisystem entities, proving the need for extensive analytical assessments of the qualitative multidimensional nature of pharmacological systems. Flexible systems must be employed to allow a comprehensive understanding of how both single-index effects and multiple signaling cascades overlap. This overlap will create potentially emergent functions that bridge the diverse elements of the intricate response systems. In general, a system is characterized as an entity that maintains its existence through the interactions between its parts [135,136]. As we have previously outlined, physiological systems can be effectively represented as a network of nodes (functional elements and vertices) connected to each other through edges, which describes the functional interactions. These functional network visualizations are referred to as graphs. While highly useful for network-based investigations, this graph-based approach unfortunately often ignores the nature and magnitude of the interactions within the network, as well as excludes potential temporal and spatial information. This creates difficulties in understanding the network activity with all its dynamic processes in different time intervals, which may be necessary for the prediction of various drug effects in vivo [137,138]. To improve the data annotation of biological networks, additional layers of information can be used to enhance the interpretative power of the node relationships. Such advancements can be seen in knowledge graphs, which depict biomedical concepts and relationships as nodes and edges [139]. Multidimensional biomedical graphs can now be constructed by integrating both human- (e.g., COSMIC [140]) and machine-curated [141,142,143] text and biomedical databases. The use of knowledge graphs can be facilitated through ML-based approaches, which will construct a low-dimensional representation of graphs to support many different applications [144]. This approach will likely preserve the graph’s local and/or global structure, while additional ML methods can help make predictions within the genomic, pharmaceutical and clinical domains [145].

While there are some clear deficiencies in the employment of graph theory to help investigate complex physiological networks, it has, however, been successfully applied in a wide variety of biomedical paradigms [146,147]. Combining graph theory from cutting-edge informatic platforms with a biomedical high-dimensionality data (e.g., GPCR receptorsome proteomics) systemic analysis will likely offer effective opportunities to explore system-level networks [148,149]. Biological changes can be set off by small variations in individual molecules (e.g., pathology-related perturbagens such as reactive oxygen species), but most frequently, they are the result of simultaneous changes in a myriad of components (e.g., multiple age-related biochemical insults) and interactions within the system [135,150,151]. It is unavoidable that human observations will miss certain cryptic biologically relevant relationships that lie within huge collections of biomedical text and data [152,153,154]. However, the discovery of cryptic relationships between drug responses and disease data in unsupervised networks can reveal unique, new pathways and potential novel drug signaling paradigms within the greater physiological network [155,156,157,158]. A network-based pathway enrichment analysis uses graph theory to find advanced functional interpretations by prioritizing topological interesting differential expression patterns in all enriched pathways [159,160,161]. Complex physiological networks are beneficial for pathway analyses of high-dimensionality datasets, since they do not follow the compartmentalized biology-based rigid structures of human/machine-curated signaling cascades. Signaling pathways, and biological pathways in general, both have a temporal and biochemical order of interactions between their components, with an upstream-to-downstream organization. Given the significant complexity of signaling cascades, it is likely that small microcircuits of signaling factors are present to regulate a stepwise control over the cascade. Additionally, interactions between multiple different cascades in the biological system can lead to overlap at varying points within other cascades. Nuanced, unsupervised pharmacological network models do not match the classically curated-signaling pathways perfectly but, however, excel in capturing the true complexity and variability of signaling transduction pathways across different levels. Such approaches therefore possess several aspects of investigation that could be vital for the inference of the GPCR-signaling pathway variation in times of temporally variant stressful perturbagen actions.

#### 2.1.2. Modulation of Networks in Disease and Aging

Aging is a pathological process that develops progressively over the lifespan of an organism. The molecular signatures of aging-related pathologies can be noted and defined at time periods less than 50% of the total lifespan of human patients [162,163]. Thus, it is likely that healthy and young individuals possess sub-disease levels of molecular pathology that are induced to grow via the accumulation of stress-induced cellular degradation. Differentiating between a state in which no overt medically observable symptoms are present (nonpathological/healthy state) and a pathological state is effected through the coordinated changes in systemic protein networks that either sense, protect or repair stress-related cellular damage [67,83,139,164,165]. Changes in this protein network could include several types of dysfunctions, e.g., both small-scale and large-scale quantitative and qualitative alterations of the protein–protein interactions [166,167]. One of the perhaps most important responsive network events is the potential change in the role of the factors termed hubs or keystones. These critical nodes demonstrate a capacity to coordinate and control a much greater number of associated factors in the network than many other nodes. Thus, in the context of aging-related pathology, hubs or keystones such as GIT2 can be changed to alter the trophic levels of network connectivity and, therefore, modulate the impact of stressful perturbations [116,168,169,170]. The human GIT protein family, comprising GIT1 and GIT2, acts as GTPase-activating proteins (GAPs) for ADP-ribosylation factor (Arf) small GTP-binding proteins [116]. Both GIT proteins were originally identified as regulators of GPCR internalization through the influence they exert on Arf GTP-binding proteins. GIT proteins are primarily considered as signaling scaffolding proteins, with their multiple domains binding to many protein partners. GIT proteins have been implicated in multiple cellular processes, including cell migration, dendritic spine formation, T-cell activation and centrosome dynamics (for review, see Reference [116]). Our research demonstrated that GIT2, compared to GIT1, possesses a potential multidimensional role in the pathological aging process, as it has been demonstrated to coordinate interactions between signaling systems that control the somatic metabolism, immune function, oxidative stress sensitivity and DNA damage responses [66,116].

In addition to dynamic reactive changes in the network functionality, the global structure of networks can be altered, as well as node connection dynamics [171]. In so doing, the network can adjust to changes the properties of either small or large numbers of factors to modulate their degree and/or betweenness status in a coordinated manner. Changes in the network that are potential causes of disease can create a pathological molecular subnetwork also called a disease module [164,165,172,173]. In addition to this module that can possibly specify the eventual generation of a discrete disease, we propose that there will likely also be stress response modules that represent sensory and reparative protein networks that engender a regulatory system above those that then dictate specific disease progression. A true system-level understanding of the formation and interplay between these disease and stress response modules and subsequent drug responses will require comprehensive classification at both the local and global levels of the topological nature of physiological networks [166,174,175,176].

The dimensional complexity of networks can be reduced into a combination of certain trophic regulatory factors. This small group of factors maintains the network integrity and allows for adaptation to deleterious and stress-related perturbations within the network [116]. The dimensional reduction of complex and intricate data is necessary to obtain the most comprehensive overview of the connection between the initial small network perturbations to the generation and network reinforcement of complex diseases. Furthermore, global structures can be remodeled into targetable therapeutic networks that are used for more specific and thorough drug investigations to develop agents that can interdict the stress-to-disease progression process that underpins pathological aging [44,45,177]. Hence, a specific aging-associated network composition will hopefully facilitate the investigation of the cause(s) and rate development of comorbidity conditions in both young and aged individuals [133,178,179]. Combining these multiple factors within a specific targeted network defines aging as a compelling risk factor for developing cardiovascular, metabolic and neurological disorders [83,180,181]. Although each disease has its own distinct end-stage clinical and pathological features, it is becoming ever clearer that many diseases likely possess a common start point, i.e., poorly repaired stress-related damage. 

Examining the disease similarity measurements with the help of differential co-expression (DCE) instead of normal differential expression has been shown to improve the definition of true common pathogenic trajectories in the network [182,183]. These common stress-related subnetworks can then be prioritized for nodes that represent effective therapeutic receptor targets that may help regulate the balance between physiology and pathophysiology [37,184]. The phrase diseasome has been codified to describe the elaborate functional network of a disease [165,185,186,187,188,189]. Akin to the creation of diseasomes, it would be facile to consider the construction of interconnected networks of proteins specifically associated with a stress sensation and response as well. Well-characterized diseasomes (either singular, comparative or multiple) have been constructed by using network-driven approaches that link diseases based on common molecular or regulatory mechanisms, such as shared genetic associations, protein interactions [190,191] or gene–disease interactions [192,193]. Similar techniques can therefore be readily applied to construct complementary stress response networks. It has been noted that certain diseases cluster together in multi-disorder diseasomes, which can contain local clusters of very similar disorders but can also reveal surprising clusters of rather heterogeneous diseases, including cardiovascular, oncological, musculoskeletal, renal and neurodegenerative conditions [5,176,191,194,195,196,197,198]. Common, underlying hidden pathomechanisms (that could potentially be enriched for stress response factors) may indeed be the reason for the clustering of apparently very different diseases. The novel information that is extracted out of the static or dynamic interacting stress–disease clusters can provide unexplored molecular information about disease phenotypes, as well as reveal new targets for drug discovery and repurposing [67,116,118,179,199,200,201,202,203,204]. Hence, the in-depth appreciation of the nuanced topological landscape features of both stress response and disease networks will likely lead to novel insights into the etiology and pathogenesis of multiple diseases [165,185,202,205,206]. The translational deconvolution of these interfaces will also help prioritize the most important GPCR-targetable disease-related/causative factors [207,208,209,210,211,212,213]. 

#### 2.1.3. The Receptors Dilemma and Network Functionality

**Figure 1:**
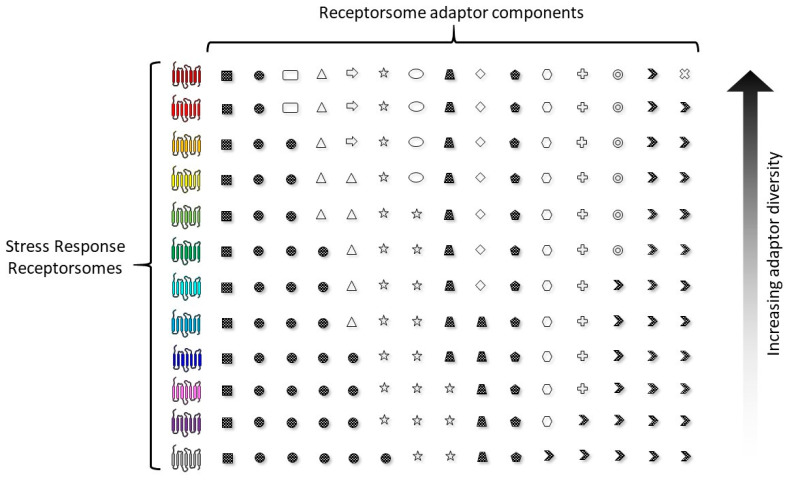
Diversity of the stress response receptorsome compositions. Here, we consider the presence of 12 hypothetical intracellular stress responsive GPCR receptorsomes (color-coded). These receptorsomes can be classified by their adaptor protein compositions. Some receptorsomes (scarlet and red) may comprise a broad variety of adaptors and, therefore, confer cellular resilience to a wide range of stressful perturbagens. Other receptorsomes (purple and grey) may consist of a smaller range of adaptors, thus reducing their potential to sense or contend with a wide range of cellular stressors. It is also noteworthy that it is likely that some receptorsome components are near-ubiquitous for all receptorsomes (e.g., square and circle); thus, these factors possess a higher trophic role in controlling multiple dimensions of stress sensation and mitigation. The adaptor proteins that are found in all of the receptorsomes are denoted using a black hashed fill. The presence of these consistent factors demonstrates that some GPCR adaptors can also function as intra-network communication factors across the diverse receptorsomes. Hence, the expression of such factors will likely serve as a network regulator function through common sensing of the receptorsome ensemble status.

**Figure 2:**
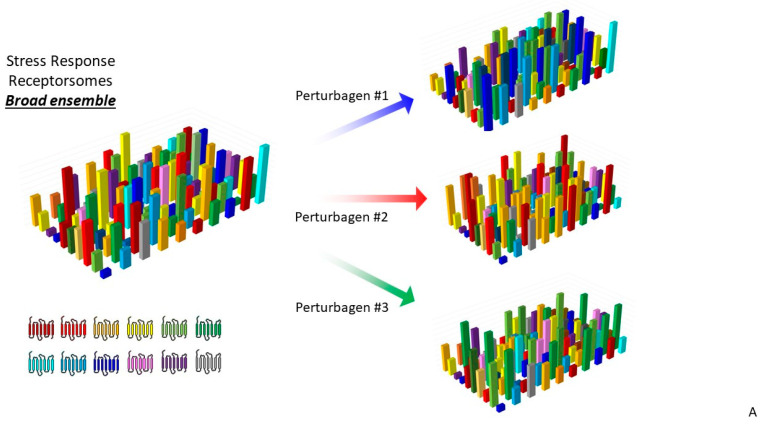

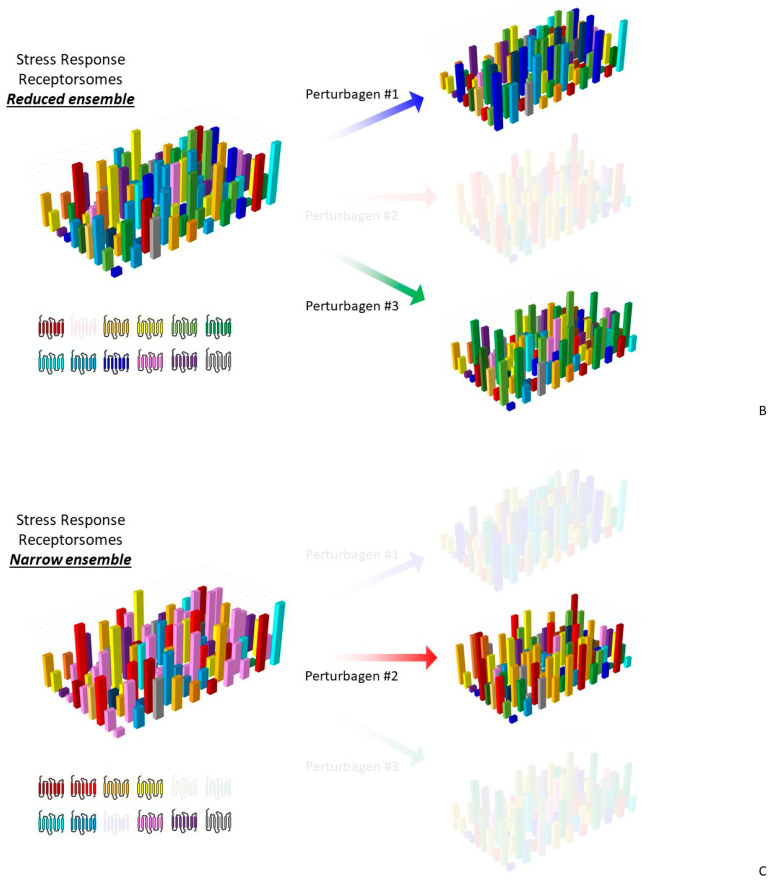
Stress response receptorsome ensemble breadth controls the cellular resilience. For each of the figure panels, a given receptorsome type is denoted by its specific color, while the vertical height of the receptorsome block represents its numerical representation in the total cellular complement of the intracellular GPCR structures. (**A**) Given a cellular scenario in which a broad variety of stress-responsive receptorsomes are present, it is likely that the dynamic responses (to distinct perturbagens 1–3), indicated by a specific elevation of the numbers of the specific sensitive receptorsome types (perturbagen 1—blue, perturbagen 2—red and perturbagen 3—green), can attenuate the damage to all three stress types. (**B**) With a reduced number of available receptorsomes (loss of red), the cell is unable to mount an effective response to one of the perturbagens (#2—red), thus resulting in an augmented level of experienced damage. (**C**) With a narrow range of receptorsomes maintained by the cell (loss of blue and green), it can only adequately contend with one stressor perturbagen (#2—red), while significant cellular damage may occur, as the majority of stressor perturbagens cannot be mitigated.

It is evident from our recent work that GPCRs can possess critical roles in stress response networks [115,133,200], e.g., Relaxin family peptide receptor 3 (RXFP3) [76]. As sensors of external tissue-to-tissue stimuli, it is therefore not surprising that an intracellular communication/sensory network for stressful perturbations to cellular functions involves these diverse and versatile signaling proteins. It is likely that this subcellular signaling network involves receptor–adaptor systems tuned to detect (on a millisecond-to-millisecond basis) harmful cellular stimuli, e.g., reactive oxygen species or high temperature stress or even prevailing levels of vital nutrients, such as free fatty acids [97,214], and then deploy responsive damage limitation and repair mechanisms. The receptor systems comprising this network will likely represent an ensemble of subcellular receptorsomes that attempt to maintain a flexible, yet optimal, range of sensitivities to a range of stressors that the cell may potentially experience in a short temporal space. It is likely that the cells that can sustain the deployment of a broad spectrum of stress-resistant complexes (Figure 1) are the most likely to survive these deleterious perturbations over a long period of time. Therefore, the cells have likely prioritized a capacity to generate efficient capacities to potentially predict the arrival of rapid insults. In this respect, it is therefore critical that a cell maintains a strong capacity to generate and coordinate the synthesis of proteins needed to construct the most effective receptor ensembles that facilitate rapid and reversible responses to the most likely range of insults a cell receives (Figure 2). 

In this context, the dilemma for sensory receptor systems within a cell is how to best deploy the resources it possesses. In this paradigm, there are several theoretical considerations that a stress sensory network needs to adjust to: (i) which types of stressful perturbations are most likely to occur to a specific cell, (ii) are there adaptor protein–GPCR relationships that develop a more efficient spectrum of resistance capacities compared to others and (iii) are there GPCR-interacting factors that can be employed to bridge multiple stress response pathways? From these issues concerning stress network construction and maintenance, it is important to consider that the stress prediction and flexibility of stressor-type resistance are crucial factors in this equation. In this context, it is vital to appreciate that the GPCR sensory network and the impinging stressors themselves exist in two distinct temporal realms, i.e., the stressors may appear at a milli/microsecond timeframe, while adjustments to the GPCR stress ensemble may take hours/days to respond to these insults. Given this disparity, it is evident that an effective stressor prediction (potentially informed by stress monitoring systems that identify the early features of failing energy metabolism) would enable the cell to not waste precious resources by creating a receptor ensemble that is not well-matched to the potential impinging stressors [215]. Coupled to this, if the cell experiences a range of stressful inputs, prioritizing the factors that facilitate between one stressor focus and the next, which may represent an important strategy for cells. Our current work with the GPCR adaptor GIT2 potentially underlines this, as GIT2 appears to possess a bridging capacity between diverse types of stress, e.g., metabolic [66,216], reactive oxygen species [216] and DNA damage [76,217]. Hence cells may therefore prioritize the creation of GIT2-associated receptorsomes in times of multiplexed stress input to ward off the accumulated damage that likely leads to age-related disease signature generation and maturation [116].

It therefore appears that an ability to interpret disease, stress sensory and therapeutic network effects may be invaluable for future system-based therapy development. Network graph quantitation and complexity measures have been studied extensively from a mathematical perspective [218,219,220]. Unfortunately, few of these specific quantitative indices deal specifically with directed graphs, i.e., graph networks that possess quantitative/functional node interactions that more closely represent the functional aspects of molecular signaling cascades. Most biological networks are depicted as directed graphs whose edges express critical interactions, flows and effective directionality [146,221,222,223]. While considerable quantitative methodologies have been employed for undirected graph networks, i.e., treewidth [220] and cycle rank [224], as well as topological indices [225], there are additional graph complexity indices such as the distance-based Wiener index [226,227,228,229,230], graph entropy measurements [231] or the Szeged index [232] that can also be computed for the more biologically relevant directed graphs. Measures for analyzing directed graphs include DAG (directed acyclic graph)-width [233,234], directed treewidth [235] and girth [236], with the latter two (treewidth and directed treewidth) being based on the game theory applied to special graph decompositions.

Game theory is the study of mathematical models of strategic interactions among rational decision-makers. The principles of game theory are employed to consider the results of model strategic situations (games) in which the choice of actions of a unitary factor or agent, and the resultant loss or benefit to that factor/agent, are affected by the choices of factors/agents [237]. Mathematical models of dynamic systems created using game theorems have been applied to gross biological phenomena such as species competition [238] and complex physiological processes such as neural network communication [239]. Game theory was developed to analyze competitions in which one factor (within a network) achieves success at the detriment of the other factor (zero-sum game). Subsequent modifications to this theory have been introduced to demonstrate potential collateral benefits of competition that were not initially apparent [240]. Game theory assists in the deconvolution of the multiple dynamic equilibria within these games (e.g., physiological or disease networks). One of the most notable examples of game theory applied to strategic equilibria in biological systems is the Nash equilibrium or so-called Prisoner’s Dilemma. In an equilibrium situation, each factor in the game has adopted a strategy that cannot improve their outcome (optimizing the gain/loss ratio), given the strategic choices of the other involved factors. Complex physiological systems, such as disease progression networks, drug-based GPCR-ligand systems and GPCR sensory networks likely consist of multiple interconnected dynamic equilibria [55]. These equilibria may represent intracellular ion release dynamics, active state receptor conversion, hypothalamic–pituitary–gonadal axis hormone feedback loops or protein–protein interactome flexibility [43,55,241].

With respect to the innate protection against aging-associated disease progression, one important molecular signaling game in this conceptual framework would be a contest between the cell’s ability to maintain an effective and efficient GPCR stress receptorsome ensemble and the random and deleterious actions of the cell stressors. In this “game”, there would be a strong component of move prediction required from both participants in the game. The two players in this game thus possess opposing goals, i.e., cell destruction/damage and cellular protection and eventual survival. As we have described before—akin to the opening gambits in chess—it is often an effective strategy to attack the opponent in a manner that is flexible and adaptable to change and reattack in a different manner. This flexibility is especially important for the GPCR players, as their responses will always tend to occur with a distinct time disadvantage compared to the stressors. This hypothetical situation highlights the importance of the capacity of the GPCR sensory network to be able to rapidly sense the onset of potential stressors as soon as possible. Here, it may be conceivable that the transcriptional machinery (linked tightly to non-G protein GPCR-signaling systems [45,242]) may form part of this sensory system, as it possesses a nuanced temporal response process that is more rapid than the primary protein turnover. In this scenario, therefore, the cell system faces a receptor dilemma in which, to gain supremacy in the cell survival game, the cells’ gambits need to be more likely to win than not. In addition to these simple cell survival games, it is also likely that such GPCR-based sensory and response networks also participate in game-related activity in the scenarios of stem cell fate decision, metabolic fuel source selection or asymmetric cargo division [66,243,244,245]. Given the success of multicellular life, it is evident that, before the onset of significant age-related pathologies (before the previously mentioned metabolic inflexion [162,163]), the stress response player tends to be successful. It is interesting to note, of course, that, with advancing age, there is a clear alteration of GIT2 expression/functionality that may demonstrate that, indeed, this is one of the key pieces deployed in the survival gambit by cells [116]. Hence, following the metabolic aging inflexion, the expression of this factor may become less than optimal [66,76,246,247] due to rises in the complexities of attacking moves made by the stress factors player.

As GPCR systems comprised of ligands (or stressful stimuli), receptors and transduction systems attempt to control the physiological homeostasis/allostasis, the components of this receptor system itself are also likely to compete to maintain, for example, the neurotransmission, endocrine axes and sensory perceptive mechanisms. Hence, we are faced with the concept of games within games, which is reminiscent of the concept of persistent homology within biological network structures [83]. Thus, systemic GPCR regulatory control over both global and cellular homeostasis is a process that is innate and vital for longevity. In this sense, a pertinent goal for effective therapeutic remediation is the support of these innate control processes. Novel therapies therefore should be designed to mimic the endogenous pattern of innate GPCR control over cellular homeostatic networks. To this end, recent research has applied game theory protocols to the network theory to accelerate the creation of so-called precision medicines [248]. Biane and coworkers developed a workflow (combining game theory and Boolean network dynamics) described as a network action game that was employed to advise the optimal drug selection. The decision-making process (for the modulation of breast cancer signaling) was modeled using game theory that defined the drug selection process among possible alternates, while Boolean networks were used to assess the interplay between the disease and drug actions on the patient homeostatic molecular system. 

The actions/strategies of the disease and drugs are often focused on edge (node-to-node connector) alterations of the specific protein–protein interactome(s). The ability to create precision medicines for a specific purpose is strongly dependent on the discovery of refined molecular signaling paradigms that selectively describe the most effective remediation route through the disease network. Farahmand et al. recently aimed to identify the crucial subnetworks within breast cancer signaling activity using a novel game theoretic approach (GTA) that integrated the use of a genome-wide expression profile and protein–protein interaction networks [249]. This approach was able to identify novel and robust metastatic markers, reveal new candidate genes for cancer susceptibility and engendered a greater feature classification performance compared to standard discriminatory models. It is also important to note that game theory approaches have also shown promise in identifying optimal peptidergic drug-like molecules with anticancer activity [250]. CGR (chaos game representation) is a method of converting a long one-dimensional sequence, e.g., text or genetic sequences, into a graphical form. CGR represents the application of nonrandom input to an iterated function system [251]. CGR has been applied (in combination with machine learning approaches such as support vector machines and deep learning) to the investigation of proteomic/peptidergic sequence analyses [250,252]. Ge and coworkers (2019) developed an advanced form of CGR (generalized chaos game representation: GCGR) to achieve a significantly higher prediction performance for the efficacy of anticancer peptide agents using public chemoinformatic databases [250]. Given these advances in game theory-based therapeutic derivation, it is evident that the application of this form of mathematical analyses has a burgeoning relevance to molecular biology and GPCR biology especially.

### 2.2. Intersection of Systemic GPCR Pharmacology with Complex Systems

The intelligent combination of multilayered integration of high-dimensionality data streams (metabolomic, transcriptomic, proteomic or epigenomic studies) is currently the optimal mechanistic way to appreciate both complex physiological systems (e.g., stress responses and aging) and GPCR-based drug responses [37,84,107,253]. There actually may be no need to increase the volume or sensitivity of data collecting systems, given our current ability to speed up drug discoveries using novel retrieval techniques to find previously cryptic data connections [47,97,247]. With the current state of knowledge in biomedical science about the complexity of systemic disease and drug responses [44,57,83,254,255,256,257,258,259,260,261,262], it is clear that therapeutic interdiction at the system level offers the best chance to combat complex and widespread diseases such as cancer, cardiovascular disease or type II diabetes mellitus (T2DM). While comprehensive high-dimensionality assessments of the complexity of the physiological/pharmacological responses have been proven to be tremendously useful, they often represent a static impression of the underlying biology, omitting the vital temporal component of complex systems [263,264,265]. This static network biology does not fit well with the fact that age-related disorders progressively grow and develop over a long timeframe. Besides that, this static approach does not intersect with the dynamic processes of therapeutic intervention. Refining and optimizing innovative therapies thus require a dynamic systems-based understanding of an individual’s underlying disease status and the mechanistic pathway of the administered drug. Therefore, for the most effective investigation of drug actions, precise temporal molecular profiles of systemic physiological activity are needed. This approach should employ an integrated pipeline that combines experiments and computational models to provide insight into how GPCR-associated stress sensor- and disease-related systems are organized through time and how this higher degree of organization leads to a functional intersection between the therapies and disease [107,266,267]. The standard biochemical estimates of the functionality of GPCR-based therapeutics typically focus on the atomic (e.g., calcium mobilization assay screening) and molecular scales (e.g., cAMP accumulation assays) in contrast to the physiology and pharmacology, which traditionally focus on the dynamic tissue/organ-level functions (e.g., plasma cortisol levels or brachial blood pressure regulation) and, more recently, on omic-related studies, (e.g., proteomic, transcriptomic, metabolomic and epigenetic assessments of drug/disease functions). The essence of modern drug design should consider how receptors sense, regulate and control the progression of age-related disorders. This pipeline should therefore aim to effectively condense data from these diverse data streams to contend with the multilevel and dynamic nature of the disease and drug effect process. 

Our current scientific ability to comprehend diseases at the system level requires the cognizance of drug discovery and development ventures that consider how specific agents can act across multiple functional networks at several dimensional states. As a result, novel GPCR-based drug discovery methods can be thought of as a network-to-network complementary matching process via which investigators are able to develop network-level pharmacological interventions, i.e., disease sensation and regulation to therapeutic matching [44,45,83,256,261,262,268]. In this context, a pertinent challenge forms: How many disease indices/outcomes must be controlled by the desired therapeutic to exert the optimal therapeutic effect with the least number of off-target actions? Using therapeutic agents that modulate the network in a subtle way might be useful to achieve the therapeutic goals, since all complex systems aim to stabilize themselves to prevent stress-inducing perturbations effectively disrupting the overall status of the network and thus generating a full disease phenotype. It is reasonable to contend that perhaps the simplest mechanism to achieve this would involve the coopting of the endogenous stress-responsive network communication systems of the body. This somatic communication system is required to allow communication between human cells but, also, between the cells and their temporally changing molecular environment. As the physiological time progresses, further levels of biochemical entropy increase, resulting in a natural degradation process that is expressed as pathological aging. The degree and rate of time-dependent degradation closely controls the development of the molecular signatures of the disease. This communication necessitates a molecular framework but, also, a mechanism for transmitting information across the cell membrane from the outside to the interior environment of the cell, of which the transmembrane heptahelical GPCR is possibly the most therapeutically relevant [1]. GPCRs facilitate communication between cells across long distances in the body, allowing for true system-level actions [6,8,269,270,271,272] and still represent the largest family of targets for the currently approved drugs [1,3,4]. In recent years, this vital long-distance regulatory communication across multiple physical/tissue and pharmacological axes [5] has been shown to not be the only physiological regulatory network of GPCRs. Hence, this somatic system is reproduced at the single-cell level—with communications being focused on coherent organelle crosstalk—by ensembles of GPCRs focused into groups that prioritize stress responses that can then be coopted to regulate the aging/disease network [2,76].

### 2.3. G Protein-Coupled Receptors as System-Level Regulators

GPCRs create intricate communication networks for an incredibly broad range of endogenous physiological stimuli. Thus, GPCRs represent effective sensors for subatomic particles like photons (rhodopsin [273]), atomic elements (calcium-sensing receptors [274]), small metabolites (succinate [275]), small molecule neurotransmitters (dopamine [276]), neuropeptide transmitters (tachykinins [28]), large glycoprotein hormones (thyrotropin [277]) and even animal-based toxins ((alpha-latrotoxins [278]). This GPCR sensory network therefore links stimuli sensations to alterations in the signaling activity via G proteins. In recent years, however, it has also been clear that GPCRs (through alternate signaling mechanisms outside specific G protein activity) also link stimuli sensations to intracellular protein expression patterns [45,76]. These distinct and multiple downstream signaling modes of GPCRs allow for the future derivation of a multiplicity of signal-selective therapies [43,279].

Given the almost ubiquitous nature of GPCRs and their functional intersections with nearly all physiological processes, it is therefore also conceivable that GPCRs can play a role in almost all drug-related mechanisms [280,281,282,283]. GPCR modulators can thus be designed to act in a synergistic manner with both large signaling networks across the body, as well as within the intracellular signaling/sensory networks. These network-level GPCR intervention strategies can then be combined with other information fluxes to produce suitable and efficient beneficial modifications of pathophysiological processes [5,133,200,242,284,285,286]. These concepts pave the way to the development of GPCR modulators that act in an endogenous pathway synergistic manner that will (i) be enhanced through the natural systemic reinforcement of endogenous axis signaling and (ii) engender only a minimal level of off-target collateral effects, as the process that is being regulated represents an evolutionarily conserved signaling axis. Therapeutic development approaches focusing on intracellular GPCR-mediated stress response network control could aid in dealing with the enormous complexity of age-dependent disease generation mechanisms, therefore enabling the faster discovery of novel drug classes that act in concert with the body’s natural damage defense and repair processes.

## 3. Pharmacological Interventions within Complex Disease Systems

A core concept within the platform of the coordinated cellular GPCR-signaling activity is the need to consider that nearly all signaling events—and proteins—are, in some relevant manner, interconnected. This level of near-ultimate connectivity requires us to appreciate how a network approach relates to the description and definition of the plastic interface between health and disease. One vital aspect of this posit is the need to consider how complex systems are (i) created to maintain balance and (ii) then are perturbed to mediate deleterious disease trajectories. Thus, we can consider the state of cellular health to be the goal of the cellular stress response network that controls the sensorial and dynamic receptor-based signaling components at the single-cell level.

During health, keystone factors such as GIT2 [116] will strive to maintain long-term stress resistance functions. Such factors likely control more dynamic and plastic regions (dynamic vertices) of the network that are sensitive to random stress perturbations. As their name implies, these network dynamic vertices can likely rapidly modulate their edge (i.e., protein–protein) interactions to counteract the stressful perturbation [81]. This concept is relatively well-known in both basic chemistry (Le Chatelier Principle) and in simplistic neuroendocrine feedback loops (e.g., activin/inhibin system in the hypothalamic–pituitary–gonadal axis [287]). However, its application in single-cell scenarios across a global lifespan introduces several important novel issues associated with receptor-based feedback systems. The concept of drug resistance is clear and well-known, i.e., repeated exposure to external therapeutics can rapidly induce tolerance and resistance [288,289,290,291]. In these circumstances, feedback within a specific system is controlled at a rapid level through receptor tachyphylaxis that may involve desensitization, alterations in receptor expression or even ligand sensitivity. With a protracted level of exposure to continuous treatment, these rapid alterations are then tolerated and incorporated into the physiological network, thus regaining stability in the presence of this consistent perturbagen [292,293,294,295]. Given this, it is likely that subcellular GPCR-based homeostatic systems are maintained in a constantly sensitive manner to both short-term and long-term perturbations and, in each case, aim to achieve balance through sensation and adaptive/protective responses. Endogenous insults such as reactive oxygen species (ROS) will likely occur sporadically and transiently over the cell’s lifespan. Repeated exposure to such an insult over a long-term scale may result in the subcellular GPCR stress response network becoming tolerant to the presence of stress/damage and then eventually try and maintain the stability of that damaged network. In this sense, the cell will then become adjusted to disease/pathobiology and no longer consider it as aberrant and distinct from the normal healthy state. The transition from healthy cells to disease ones is thus likely to coincide perhaps with the metabolic aging inflexion point [162,163]. Therefore, disease progression (which may indeed have a common route in pathological aging [116]) may be a function of subcellular stress network sensitivity, flexibility and stability. Considering this hypothesis, it would be important to reevaluate how we can classify and identify what constitutes a healthy and a pathological network status within a cell. To this end, biomarkers of network efficiency or cellular health need to be developed—researchers have already begun this process, especially with respect to aging and epigenetic control [66,296,297]. Hence, with respect to this novel form of disease trajectory modulation, it is important to appreciate and dissect how the network complexity and plasticity can be associated with such imbalance effects. In the following sections, we shall discuss how GPCR-based sensory systems can be adapted to regulate such higher-order network functions.

### 3.1. Homeostasis and Allostasis within Networks—The Role of GPCRs

As we have discussed previously, the GPCR sensory network likely can control both long-term and short-term stressful events at the single-cell level across the lifespan. Complex biological events, at the network level, will therefore demonstrate two distinct types of activity, i.e., homeostasis (long-term global regulation) and allostasis (short-term local network maintenance, e.g., dynamic vertex rearrangement). Multiple concepts of allostasis were derived from the work of Sterling and Eyer (1988) [298]. They defined allostasis using the following description: an organism must vary all the parameters of its internal milieu and match them appropriately to environmental demands. For the context of health network disruption, characterized by the persistence of perceived (e.g., disruption of effective glucose metabolism) or actual (oxygen radical molecular damage) insults, the cellular stress response network needs to maintain consistent vigilance and self-checking activity. This ongoing activity can often involve considerable molecular energetic behaviors and, thus, present a significant burden itself, e.g., elevation of the default network activity in the brain has been associated with increased risks for neurodegeneration [299]. Hence, the dynamic plasticity of physiological networks, often described as the process of allostasis, facilitates the maintenance of global longer-term homeostasis. As we have previously discussed, potential failures in this process can represent the first foothold of disease patterns within the network [107,300,301,302].

In situations of cellular strain and stress, molecular allostasis likely ensures stability through perturbagen-induced changes by modifying the setpoints and parameters of the feedback control [303,304]. Indeed, such functional predictive stress response behaviors have also been recently shown with respect to lifespan regulation and oxidative burdens in a gender-distinct manner [247]. Despite being a basically beneficial reaction, allostasis may also expose the cell to a new kind of strain referred to as an allostatic load, which may result in an even critical loss of cellular viability. To help understand how such burdens of anticipatory allostasis can perturb stress response networks, a recent new addition to Sterling’s original concepts was introduced by Lee (2019) [305], i.e., the Paradigm of Allostatic Orchestration (PAO). The PAO represents a conceptual understanding of how neural (brain) inputs into the homeostatic network can facilitate the creation of an active allostatic state. In a similar vein, the concept of interoception conceptualizes the sequelae of the reverse sequelae of this information stream, i.e., the psychosocial/cognitive effects of physiological homeostasis [306,307].

Lee (2019) proposed that an allostatic state represents a neutrally focused monitoring mechanism that, upon global network integration, generates the entity of somatic homeostasis [305]. The physiological nature and efficiency of the somatic allostatic state has been proposed to underpin multiple disorders associated with ongoing stressful perturbations/insults, such as chronic pain [308], immune and thyroid dysfunction [309,310], irritable bowel syndrome [311] and stimulant addiction [312]. The allostatic state proposed in the PAO does not apply to pathology or disease alone, nor does it suggest that controlling the neural effects (or stressors) are the sole cause of any disorder; rather, it serves to reinforce (like interoception) that there is always a bidirectional influence between any given system expression and the functionality of the network-controlling features. The PAO also addresses the generation of the potential damage-generating locus within systemic networks, i.e., allostatic load or burden. The need to maintain this network surveillance is likely to generate a significant stress upon any cellular health network and, thus, could be key to its gradual dysfunction over the lifespan. Hence, this novel concept within the PAO is optimal anticipatory oscillations. This aspect of the PAO is effectively the same as Sterling’s definition of health as the optimal predictive fluctuation. Optimal anticipatory oscillation builds on an appreciation of the network controlling systems as “prediction machines” [313,314], and reflects the capacity for matching between operations in the cellular network with the typically oscillatory features of the cells in the context of the whole organism in ways that reduce the limitations, enhance resilience or expand the range of possible functionalities or opportunities. For example, with respect to cardiovascular activity, optimal anticipatory oscillation may be demonstrated as the heart rate variability, indicating a capacity for rapid recalibrations of the cardiac output in context-sensitive ways that result in a decreased risk of morbidity or mortality [315,316].

In a similar manner, recent research has also shown that, with continuous glucose monitoring (CGM), humans often display considerable diversity in their ability to modulate elevations in postprandial glucose elevations [317]. These diverse gluco types shown by the patients may also represent distinct protective or resilient states of the patients to the systemic glucose perturbations. Innate optimal anticipatory oscillation may be associated with characteristic sleep patterns [318], motor behaviors or sensory acuity [319] or positive cognitive appraisals [320]. It is interesting to note that circadian clock regulation likely underpins many of these network influencers, which again reinforces the importance of molecular aging pathways in disease etiology, as both circadian clock proteins and DNA damage repair (DDR) proteins have coevolved [83,321]. The synergy between these two short-loop allostatic systems (clock and DDR) likely provides a form of cellular damage feedback to time perception that can then further inform the allostatic control of the health network. In this specific example, a molecular characterization of the allostatic state facets of optimal anticipatory oscillation (that provides a positive health benefit across the organ systems) may lead to the creation of nuanced therapeutics that reduce the prevalence and incidence of morbidity and confer systemic allostatic resilience. To this end, our recent work linking the aging controller GIT2 to systemic cellular resilience via its coordination of DDR, oxidative stress resistance and circadian clock control via a functional engagement of the RXFP3 receptor provides an interesting novel route of system-level drug developments [76,133]. With regards to the RXFP3-GIT2 system, it appears that this allostatic sensory network maintains an observation of potential stress/damage through a process balancing energy source usage, oxidative radical management and DNA repair [66,76,217]. Such investigations indeed may demonstrate the future benefits of therapeutics targeted towards the points of allostatic control and integration within the health network as opposed to controllers of overt disease symptoms.

### 3.2. Disease Signatures at the Subcellular Level

The ability to identify and classify diseases at the molecular/cellular level before the development of perceptible symptoms will offer an important locus for novel prophylactic therapeutics. Cellular health and dysfunction can be considered as simply different network states. Hence, an agent that could alter a disease network signature at an early stage may potentially be described as a disease trajectory modulator, as opposed to remedial therapeutics. For the future development of these trajectory modulators, it is of the utmost importance that effective technologies for the identification and characterization of such molecular disease signatures at the single-cell level are developed. The early-stage identification of characteristic disease signatures is important, because, in these early stages, there will probably be minimal cellular pathology masking the molecular signature, whereas, in later stages, many network perturbations may be due to cellular degradation rather than disease etiology. In addition, network perturbations at an early stage are likely to be of smaller magnitude, potentially facilitating their reversal with only a modest efficacy of a targeted therapeutic agent. The persistent homology of the stress response network structure is likely to be preserved across multiple scales of magnitude. Therefore, techniques such as topological data analysis (TDA) [83,84,322] can be used to capture the important characteristics of a mature and complex disease already at an early temporal stage when only small-magnitude perturbations may exist. The input of high-quality high-dimensionality data is crucial for dimensional condensation approaches such as TDA [247], while the effective integration of such data will be necessary, as cellular dysfunction and disease signatures are likely to occur in a multiscale manner [107]. Recently, diagnostic and therapeutic molecular disease signatures for many different conditions have been proposed, e.g., diabetes mellitus [317], sporadic inclusion body myositis [323], breast and prostate cancer [324,325], pathological aging [179,326] and immunosenescence [327,328]. The capacity to accurately measure specific cellular dysfunctions and predict the future phenotype and outcomes of various treatment options are necessary for the potential success of tailoring therapies for individual patients with their unique molecular disease networks [84]. Molecular disease signatures can involve contributions of many genes/proteins and may be extremely complex even at the early stages. Various studies have reported important developments in informatics deconvolution approaches related to the derivation of multifactorial disease signatures [329,330,331]. Given that individual cellular responses to imping stressors will subsequently spread to neighboring cells [332,333], it is evident that a more nuanced understanding of how disease signatures are created from single-cellular perturbation responses will be vital for the future development of trajectory modifiers for aging-associated disorders.

### 3.3. Precision GPCR Interventions for Complex Systems

Traditionally, pharmacological compounds are identified and prioritized based on their biological effects through modulation of the activity of a unitary target, e.g., a specific enzyme, the gating of an ion channel or the stimulation of a certain receptor. Such monolithic target focusing does not consider the likelihood that cellular and somatic functions are the result of a myriad of signaling systems that are connected to each other. The routine implementation of high-dimensionality data acquisition and analysis has now made it possible to not only understand the complexity of systemic diseases but, also, to understand the possibility of interindividual variations in the etiology of diseases, which can be specifically addressed with what is termed precision, personalized or individualized medicines. Precision medicines [107,334,335,336] possess specific efficacy profiles, which are selected to match best with the specific disease profile of the patient. In this emerging medical model, computational medicine plays an important role for assisting physicians in their decision-making [337] and stratification [323] by combining data analysis and systems biology modeling. In the field of precision medicine, the assessment of a drug efficiency necessitates the ability to identify the various disturbances in a healthy system network at both the somatic and single-cellular level that are caused by a disease process. In previous sections, we discussed that, with respect to subcellular stress responses, GPCRs are among the most tractable therapeutic systems to control individual variations in these resilience networks. Therefore, in the context of precision medicine, we should consider which strategies to implement for drug development: the highly inefficient drug design for individual patients or the drug design for groups of patients clustered together in a way that is already tractable to GPCR therapy [338]. The curation of known signaling paradigms and their enrichments across the specific networks of diverse patient groups can help to achieve this second option. First, the high-dimensionality profiling of individual patients is crucial. Specific GPCR-sensitive signaling cascades can then be extracted out of these datasets [57]. We previously mentioned a problem with such a strategy, namely the diversity of signaling at the level of a single GPCR unit due to their pluri-dimensional signaling profiles. Therefore, it can be argued that distinct patterns of GPCR adaptor coupling—engineered to create a diverse response range to cellular stressors—could be associated with different subforms of diseases or even different populations of patients. The extensive acquisition of high-dimensionality data from numerous patients will be necessary to confirm this. Existing GPCR-based therapies have already shown a clear capacity to facilitate multiple drug interventions for distinct patient populations via their ability to differentially control distinct patterns of downstream signaling. GPCR-based agents exhibiting selectivity for signaling via GIT2 [76], NHERF [339] or β-arrestin [29,44,57] may prove differently effective in various patient groups of which the pathology is related to such signaling dissimilarities. A single GPCR target can functionally interact with multiple downstream signaling adaptors within an individual in many distinct cell background situations. Therefore, these transmembrane receptors provide a desirable opportunity to produce highly tailored efficacy profiles specific for processes, tissues, patients and disease clusters [23,43,73,340]. Recently, it has also been shown that the regions encoding GPCRs are often specific loci for disease initiation via somatic mutations that will probably also exist in a patient cluster-based manner [341,342,343,344,345]. These features of GPCR biology should therefore drive the pioneering of GPCR-based therapies with pluri-dimensional efficacy, both within a classical cell surface and subcellular stress-responsive manner, as perhaps one of the most convenient and potentially effective forms of precision medicine [23,44,45].

## 4. Summary

It is clear from multiple lines of evidence that the molecular signatures of aging, at the single-cell level, are some of the most potent triggers of diseases (cancer, T2DM, chronic kidney disease, chronic obstructive pulmonary disease and cardiomyopathy) that result in nearly 80% of global mortality [346,347,348]. Here, we have discussed how, in addition to their role as cell surface sensors of soluble factors, subcellular functions of GPCRs associated with stress responses likely control the connectivity between classical cellular perturbations and the incipient molecular signatures of diseases. This concept suggests the presence of a novel form of a pharmacological system that represents a highly nuanced functional interface between healthy stress response networks and pathological disease-generating molecular signatures. 

While this current posit is in a relatively early conceptual stage, it is important however to develop testable and quantifiable frameworks that can be employed in the future to allow the application of standard statistical methods to refine the therapeutic developments. In this context, it may be useful to develop a workable GPCR meta-information environment. Hence, multiple diverse streams of GPCR data, e.g., quantified receptor expression (mass spectral counting), adaptor protein expression, stoichiometric interactome constituents, transcriptomics and metabolic products, can be simultaneously assessed to create a single gestalt readout for comparative studies. This GPCR meta-information can also be assessed through the lens of subcellular compartments or even distinct intracellular liquid phases [349]. This level of refinement will hopefully allow some degree of nuance with respect to the stratification of distinct receptorsome ensembles. This quantitative data can then be subject to a classical signaling pathway analysis, as well as a natural language processing concept investigation, to yield functionally relevant profiles in these different compartments in response to aging-linked stress perturbations. To contend with this considerable corpus of data, these diverse analytical streams can be hyper-annotated to the surface of TDA structures that allow the investigation of data/text/pathway correlations in both small local structural features as well as at a higher data structure level [83,322,324]. With consistent applications of this potential pipeline to distinct pathophysiological scenarios, the quantitative analyses of these GPCR sensory systems will hopefully become a routine component of therapeutic development strategies.

With the enhanced definition of multiple distinct GPCR signaling profiles, it is hoped that this will enable researchers to create medications that possess specific efficacy profiles tailored to personalized disease states. To engineer a feasible future application of high-dimensionality data-based precision medicines, it is first crucial for the therapeutic designer to generate an in-depth molecular characterization of the disease target itself. This appreciation can first take the form of a data integration point, e.g., using a topological composite mechanism such as Plurigon or Iris [322,324]. The overlaying of multi-omic disease characteristics upon an annotated data structure [107] will create a testing space for the interrogation of this construct with similar analytics based upon the therapeutic drug response signatures generated using either previously existing data (GEO signatures—https://www.ncbi.nlm.nih.gov/geo/, PRIDE—https://www.ebi.ac.uk/pride/ or the Human Metabolome database—https://hmdb.ca/, accessed on 24 November 2021) or with *de novo* laboratory-generated high-dimensionality drug response data. This intense level of data correlation will help highlight the most tractable points of molecular intervention of the prioritized therapeutics for a specific disease indication. Given the potential efficacy of this process, it is, however, critical that this convoluted process be refined to allow for rapid clinician-based patient stratification of the disease and, then, for efficient therapeutic selection to most effectively treat a specific patient group. From the multiplexed disease signatures, the most reliable disease-characterizing and drug-tractable protein groups are likely to form a specific subnetwork. These factors can then be rapidly screened for using patient plasma or circulating peripheral blood mononuclear cells applied to dedicated glass microplates spotted with antibodies for high-priority disease-characterizing and therapeutically tractable protein sets. This data will then be able to determine the presence and relative severity level of the specific disease for which the antibody chip was created. The specific protein expression patterns, potentially 50–100 proteins, will then be cross-matched with the previously mentioned high-dimensionality drug signatures that have been pre-assessed against the specific disease paradigm. This process will therefore help prioritize what potential intervention possesses the greatest capacity to remedially regulate the disease signatures identified from the initial patient materials. This overall paradigm therefore uses rapid database searching and prioritizing to both refine the disease signature presence/severity and then suggest to the clinician what type of intervention may be best-suited to the aspects of the disease network that are the most prominent in a specific patient. In this manner, the precision of the intervention is based upon the actual molecular profile of the patient. Thus, the delivery of precision medicine here is based upon the recognition that both the disease and therapeutic response exit ostensibly at a high-dimensional level of complexity.

Our proposed GPCR stress response network will likely serve to control the allostatic balance first at the single-cellular level and then across multiple cell groups, tissues and, ultimately, at the whole organismal level. In this sense, this concept presents a new therapeutic realm to possibly control the development of diseases at the single-cell level many decades before the generation of clinically identifiable symptoms. The ultimate capacity to understand and regulate this system could be paradigm-shifting from a global health perspective.

## Data Availability

Data sharing not applicable No new data were created or analyzed in this study. Data sharing is not applicable to this article.
